# Peripheral Substitution: An Easy Way to Tuning the Magnetic Behavior of Tetrakis(phthalocyaninato) Dysprosium(III) SMMs

**DOI:** 10.1038/srep08838

**Published:** 2015-03-06

**Authors:** Hong Shang, Suyuan Zeng, Hailong Wang, Jianmin Dou, Jianzhuang Jiang

**Affiliations:** 1 Beijing Key Laboratory for Science and Application of Functional Molecular and Crystalline Materials, Department of Chemistry, University of Science and Technology Beijing, Beijing 100083, China; 2 Department of Chemistry, Liaocheng University, Liaocheng 252059, China

## Abstract

Two tetrakis(phthalocyaninato) dysprosium(III)-cadmium(II) single-molecule magnets (SMMs) with different extent of phthalocyanine peripheral substitution and therefore different coordination geometry for the Dy ions were revealed to exhibit different SMM behavior, providing an easy way to tuning and controlling the molecular structure and in turn the magnetic properties of tetrakis(tetrapyrrole) lanthanide SMMs *through* simple tetrapyrrole peripheral substitution.

In addition to their interesting organic semiconducting properties^1^,^2^,^3^,^4^,^5^, the magnetic properties of sandwich-type multiple(phthalocyaninato) lanthanide complexes have aroused increasing research interests in recent years due to their significant importance in high-density storage devices, spintronics, and quantum computations^6^,^7^,^8^,^9^. However, most efforts paid in this direction seem to be focused on the double-decker SMMs with the aim of clarifying the structure-property relationship because of their easy synthesis and well defined molecular structure^10^,^11^,^12^,^13^,^14^,^15^,^16^,^17^,^18^. In close relationship with molecular magnet engineering, very lately the bis(phthalocyaninato) lanthanide double-decker as intrinsic SMM scaffold has been fabricated into multiple-decker, in particular quadruple-decker sandwich compounds, and their molecular magnetic properties have also been investigated in a preliminary manner^19^,^20^,^21^. In 2011, the SMM nature of {[Pc(OC_4_H_9_)_8_]Dy[Pc(OC_4_H_9_)_8_]Cd[Pc(OC_4_H_9_)_8_]Dy[Pc(OC_4_H_9_)_8_]} was first revealed by our group^19^. A little later, a series of {(Pc)M_1_(Pc)Cd[Pc(OC_4_H_9_)_8_]M_2_[Pc(OC_4_H_9_)_8_]} (M_1_-Cd-M_2_ = Tb-Cd-Tb, Tb-Cd-Y, Y-Cd-Tb) SMMs were designed and synthesized for the purpose of clarifying the effect of long range f-f interaction between the two lanthanide ions separated by a diamagnetic ion on the magnetic properties^20^. In addition, another series of homo- and hetero-nuclear quadruple-decker phthalocyanine analogues (Pc)M(Pc)Cd(Pc)M(Pc) and (Pc)Y(Pc)Cd(Pc)M(Pc) (M = Tb, Dy, Er) have also been prepared with their magnetic properties comparatively studied, confirming the long-range interactions between two f-electronic centers^22^. However, the effect of coordination geometry for the lanthanide ions sandwiched between phthalocyanines on the magnetic properties of this novel series of particular quadruple-decker SMMs still remains unexplored due to the lack of effective method in tuning and controlling the quadruple-decker molecular structure, to the best of our knowledge.

## Results and Discussion

**Figure 1:**
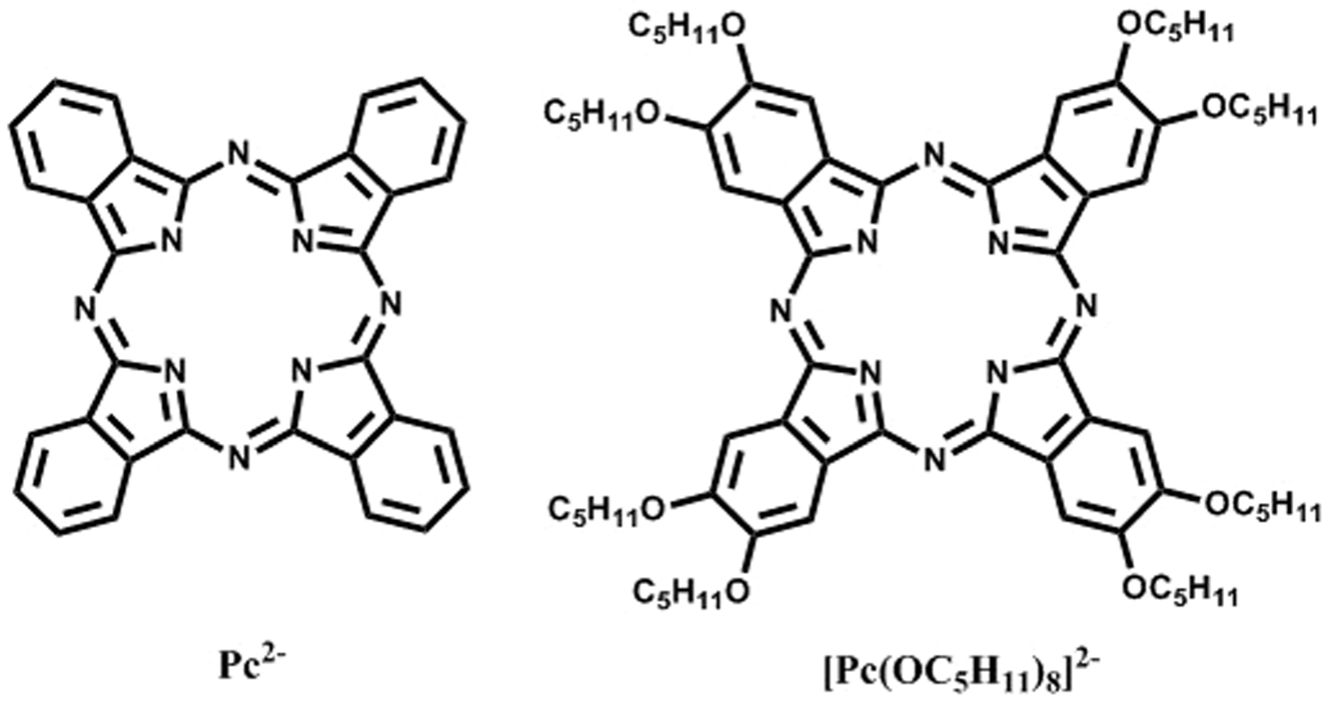
Schematic structures of Pc^2−^ and [Pc(OC_5_H_11_)]^2−^.

In the present paper, with the aim of tuning and controlling the molecular structure (actually the coordination geometry of the dysprosium spin carrier), two new tetrakis(phthalocyaninato) metal quadruple-decker complexes {(Pc)Dy[Pc(OC_5_H_11_)_8_]Cd[Pc(OC_5_H_11_)_8_]Dy(Pc)} (**1**) and {[Pc(OC_5_H_11_)_8_]Dy[Pc(OC_5_H_11_)_8_]Cd[Pc(OC_5_H_11_)_8_]Dy[Pc(OC_5_H_11_)_8_]} (**2**) {H_2_Pc = unsubstituted phthalocyanine; H_2_Pc(OC_5_H_11_)_8_ = 2,3,9,10,16,17,23,24-octakis(pentyloxy)phthalocyanine} with different extent of peripheral substitution for the phthalocyanine ligands have been designed and prepared from corresponding neutral bis(phthalocyaninato) dysprosium double-decker {(Pc)Dy[Pc(OC_5_H_11_)_8_]} or {[Pc(OC_5_H_11_)_8_]Dy[Pc(OC_5_H_11_)_8_]}, respectively, Figure 1. Single crystal X-ray diffraction analysis clearly reveals the different skew angle (defined as the rotation angle of one ring away from the eclipsed conformation of the two rings) for the bis(phthalocyaninato) dysprosium unit in these two quadruple-decker compounds. This in turn results in their obvious different SMM behavior according to magnetic measurements, not only revealing the structure-magnetic property relationship but more importantly providing an easy but effective way towards effectively tuning the SMM behavior of tetrakis(phthalocyaninato) lanthanide quadruple-deckers *through* simple peripheral substitution.

On the basis of theoretical rationalization, tetrakis(phthalocyaninato) rare earth-cadmium complexes {[Pc(OC_8_H_17_)_8_]M[Pc(OC_8_H_17_)_8_]Cd[Pc(OC_8_H_17_)_8_]M[Pc(OC_8_H_17_)_8_]} (M = Y, Pr-Yb except Pm) have been effectively synthesized and isolated quite recently *through* the solution instead of solid phase condensation pathway with neutral instead of reduced form of bis(phthalocyaninato) rare earth double-decker as starting material^19^,^20^,^21^. In the present case, both bis(phthalocyaninato) dysprosium double-decker compounds {(Pc)Dy[Pc(OC_5_H_11_)_8_]} or {[Pc(OC_5_H_11_)_8_]Dy[Pc(OC_5_H_11_)_8_]} were employed for the synthesis of target tetrakis(phthalocyaninato) dysprosium-cadmium complexes, leading to the isolation of {(Pc)Dy[Pc(OC_5_H_11_)_8_]Cd[Pc(OC_5_H_11_)_8_]Dy(Pc)} (**1**) and {[Pc(OC_5_H_11_)_8_]Dy[Pc(OC_5_H_11_)_8_]Cd[Pc(OC_5_H_11_)_8_]Dy[Pc(OC_5_H_11_)_8_]} (**2**) with good yield of 73% and 61%. It is worth noting that when the heteroleptic bis(phthalocyaninato) dysprosium {(Pc)Dy[Pc(OC_5_H_11_)_8_]} was employed as starting material, only quadruple-decker with the conformation {(Pc)Dy[Pc(OC_5_H_11_)_8_]Cd[Pc(OC_5_H_11_)_8_]Dy(Pc)} (**1**) was isolated without the detection of other possible quadruple-decker isomers according to the NMR spectroscopy^23^,^24^,^25^, due to the dominant electronic effect in the heteroleptic double-decker molecule of {(Pc)M[Pc(OC_5_H_11_)_8_]}.

Figure S1 and S2 (Supplemental Information) show the ^1^H NMR and ^1^H-^1^H COSY spectra of **1** and **2**, respectively. Assignments of all the signals for both quadruple-decker compounds were easily achieved by virtue of the sets of chemical shift in the Pc(OC_5_H_11_)_8_ ligand with the help of two dimensional ^1^H-^1^H COSY spectra, Table S2 (Supplemental Information). It is worth noting that for the quadruple-decker {(Pc)Dy[Pc(OC_5_H_11_)_8_]Cd[Pc(OC_5_H_11_)_8_]Dy(Pc)} (**1**), the nonperipheral protons of the inner Pc(OC_5_H_11_)_8_ rings give signal at *δ* −38.55 ppm. Very interestingly, despite the replacement of the outer unsubstituted Pc rings by Pc(OC_5_H_11_)_8_ in {[Pc(OC_5_H_11_)_8_]Dy[Pc(OC_5_H_11_)_8_]Cd[Pc(OC_5_H_11_)_8_]Dy[Pc(OC_5_H_11_)_8_]} (**2**), the nonperipheral protons of the inner Pc(OC_5_H_11_)_8_ rings still resonates almost at the same position, *δ* −38.05 ppm, revealing their same axial anisotropy^26^,^27^ and suggesting the almost same skew angle for the (Pc)Dy[Pc(OC_5_H_11_)_8_] subunit in **1** and [Pc(OC_5_H_11_)_8_]Dy[Pc(OC_5_H_11_)_8_] subunit in **2** in *solution* state.

**Figure 2:**
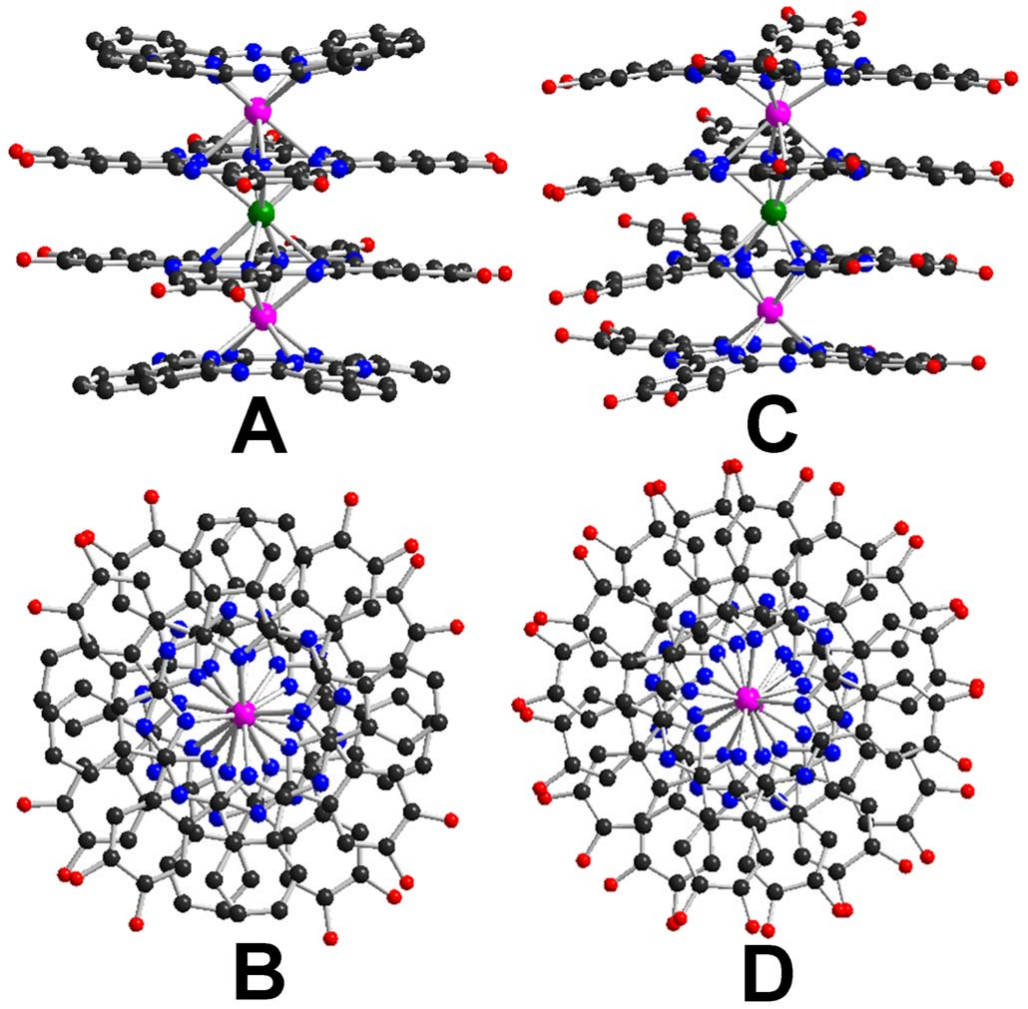
Molecular structures of 1 (A, B) and 2 (C, D) in side view and top view with all hydrogen atoms and C_5_H_11_ side chains omitted for clarity [Dy(III) pink, Cd(II) green, C black, N blue, and O red].

Single crystals of both quadruple-decker compounds suitable for X-ray diffraction analysis were obtained by diffusing methanol into the solution of corresponding compound in CHCl_3_. Compound **1** crystallizes in the monoclinic system with a *C*2/c, while **2** in the triclinic system with a *P*-1 space group. The sandwich nature of both tetrakis(phthalocyaninato) dysprosium(III)-cadmium(II) compounds with symmetric quadruple-decker molecular structure was confirmed by single crystal X-ray diffraction analysis, Table S3 (Supplemental Information). As shown in Figure 2, each quadruple-decker molecule of **1** is composed of two {(Pc)Dy[Pc(OC_5_H_11_)_8_]} units connected by an intermediate Cd(II) ion which is eight-coordinated by eight isoindole nitrogen atoms from two inner phthalocyanine ligands. The skew angle for the {(Pc)Dy[Pc(OC_5_H_11_)_8_]} subunit is 41.42° and the magic angle (defined as the angle between the S_8_ axis and a Dy-N direction) is 53.05°, Table S4 (Supplemental Information), revealing the almost ideal square-antiprismatic (SAP) polyhedron around the dysprosium ion (45° for the skew angle and 54.74° for the magic angle)^28^. The displacements of the dysprosium ion with respect to the four isoindole nitrogen atom mean planes are Dy-N_4_(Pc) = 1.326 Å and Dy-N_4_[Pc(OC_5_H_11_)_8_)] = 1.621 Å. As for the homoleptic analogue **2**, the skew angle for the {[Pc(OC_5_H_11_)_8_]Dy[Pc(OC_5_H_11_)_8_]} subunit decreases to 23.46°, resulting in a significantly distorted square antiprism coordination polyhedron around the dysprosium ion, while the magic angle is 52.59°. The displacements of the dysprosium ion with respect to the four isoindole nitrogen atom mean planes are Dy-N_4_[Pc(OC_5_H_11_)_8_)] (outer) = 1.321 Å and Dy-N_4_[Pc(OC_5_H_11_)_8_)](inner) = 1.681 Å, respectively.

The static magnetic properties of the two tetrakis(phthalocyaninato) dysprosium quadruple-deckers have been investigated. The temperature dependence of the magnetic susceptibility *χ*_M_*T* for **1** and **2** is shown in Figure S3 (Supplemental Information). The values of *χ*_M_*T* at 300 K are 28.32 for **1** and 28.04 cm^3^ K mol^−1^ for **2**, respectively, both of which are close to 28.34 cm^3^ K mol^−1^ that is expected for two Dy(III) ions [^6^*H*_15/2_, *S* = 5/2, *L* = 5, *g* = 4/3]^29^,^30^,^31^. When the temperature gets lowered, the *χ*_M_*T* values decrease slowly until about 60 K, then decrease quickly to a minimum value of 23.32 and 22.02 cm^3^ K mol^−1^ at 2 K. Such kind of magnetic behavior for both compounds should mainly originate from the crystal-field effect including thermal depopulation of the dysprosium(III) Stark sublevels and the presence of antiferromagnetic dipole-dipole interaction between the two adjacent double-decker subunits.

According to Curie-Weiss law, fitting the experimental data from 2 to 300 K gives the Curie constant (*C*), 28.38 and 28.74 cm^3^ K mol^−1^ for **1** and **2**, respectively, and Weiss constant *θ* of −5.89 (**1**) and −6.56 K (**2**). Such a fact that the field dependence magnetization *M* (*H*/*T*) data at low temperature are far from the saturation magnetization value of 10 μ_B_ expected for even one Dy(III) ion (*J* = 15/2, *g* = 4/3), in combination with the non-superimposition character of the isothermal field dependence *M* vs *H/T* curves for **1** and **2**, Figure S4 (Supplemental Information), discloses the presence of the crystal-field effect and the magnetic anisotropy for the Dy(III) ion in the quadruple-decker compounds, suggesting their potential SMM nature^29^,^30^,^31^,^32^,^33^,^34^,^35^,^36^,^37^,^38^.

**Figure 3:**
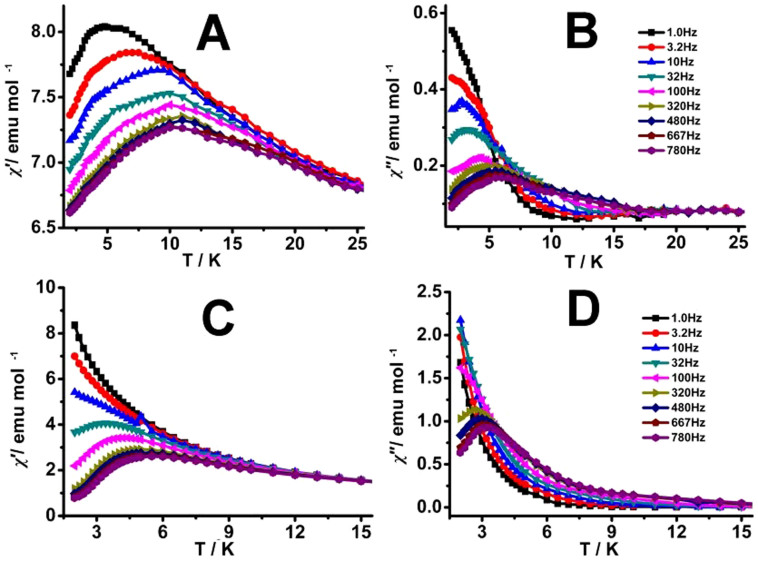
Temperature dependence of the in-phase (*χ*′; A, C) and out-of-phase (*χ*″; B, D) ac susceptibility of 1 (A, B) and 2 (C, D) under zero applied dc field.

For the purpose to further reveal the magnetic relaxation of these compounds, the dynamics of magnetization was studied on multicrystalline powder samples of **1** and **2** in a 3.0 Oe ac field oscillating at 1.0–780 Hz. Figure 3 displays the plots of *χ*′ *vs. T* *χ*″ *vs. T* in a zero dc field for **1** (A, B) and **2** (C, D). As can be seen, the frequency dependent in-phase (*χ*′) and out-of-phase signals (*χ*″) show the slow relaxation of magnetization for the two compounds, confirming their SMM nature. Nevertheless, for **1**, the *χ*″ signal starts to show clear frequency-dependent peak at the frequency as even low as 10 Hz, while **2** shows *χ*″ peak at 320 Hz, revealing a faster relaxation due to the quantum tunneling of magnetization (QTM) than **1** because of the larger deviation of the coordination geometry for the Dy spin carriers from the ideal square antiprism molecular symmetry as indicated by the skew angle of 23.46° for **2**, in comparison with the skew angle of 41.42° for **1**. Based on a thermally activated mechanism, *τ* = *τ*_0_exp(*U*_eff_/*kT*) and *τ* = 1/(2π*ν*), the Arrhenius law fitting for the picked peaks in the *χ*″ *vs* *T* curves in zero field for these two compounds was then carried out, revealing a linear relationship between ln(*τ*) and 1/*T* in the temperature range of 2.5–6.0 K for **1** and 2.5–3.2 K for **2**. This in turn results in an energy barrier with *U*_eff_ = 16.42 cm^−1^ (23.65 K) and pre-exponential factor τ_0_ = 3.84 × 10^−6^ s with *R* = 0.996 for **1**, Figure S5 (Supplemental Information), and *U*_eff_ = 12.00 cm^−1^ (17.28 K) and *τ*_0_ = 8.83 × 10^−7^ s with *R* = 0.999 for **2**. Obviously, the energy barrier of **1** is larger than that of **2**, revealing again the effect of the deviation of the coordination geometry for the dysprosium spin carriers from the ideal SAP polyhedron on the magnetic properties of sandwich-type quadruple-decker complexes^28^. As detailed above, unlike in the solution state, **1** and **2** in the single crystal state possess two similar magic angle but significantly different skew angle for the (Pc′)Dy(Pc′) units, which then results in the different SAP environment. This in turn is responsible for the different energy barrier between these two compounds. Nevertheless, graphical representation of *χ*″ versus *χ*′ (Cole-Cole plot) at 2.0, 3.0, 5.0 K for both **1** and **2** give one semicircle, suggesting the existence of one magnetic relaxation processes, Figure S6 (Supplemental Information). Fitting of the experimental data according to the modified Debye function equation^39^ gives the following sets of parameters with α = 0.19–0.29 for **1** and α = 0.24–0.25 for **2**.

**Figure 4:**
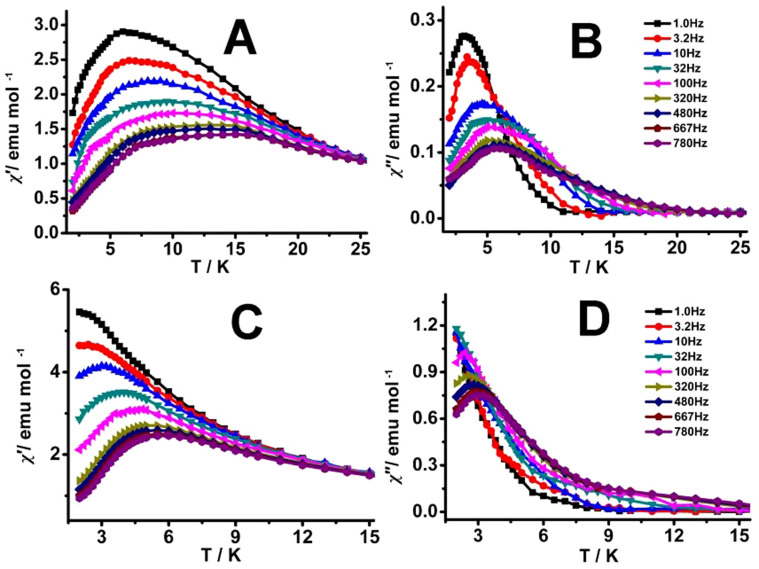
Temperature dependence of the in-phase (*χ*′; A, C) and out-of-phase (*χ*″; B, D) ac susceptibility of 1 (A, B) and 2 (C, D) under 2000 Oe applied dc fields.

In addition, the dynamic susceptibility was also measured in a static magnetic field *H* = 2000 Oe to suppress the QTM for both compounds. As exhibited in Figure 4, very clear peak is observed in the *χ*″ vs *T* curves of **1** even at the frequency as low as 1.0 Hz, while the peaks for **2** are able to be observed only above relatively high frequency by 100 Hz. Anyway, these results indicate a typical slowing down of the relaxation mechanism. Nevertheless, the ac susceptibility data for these two compounds show an overall reduction in height due to the saturation effect. The corresponding Arrhenius law fitting for the *χ*″ *vs* *T* data under an external 2000 Oe field gives effective energy barrier *U*_eff_ = 27.35 cm^−1^ (39.38 K) and τ_0_ = 7.91 × 10^−7^ s for **1** and *U*_eff_ = 15.23 cm^−1^ (21.93 K) and τ_0_ = 1.28 × 10^−7^ s for **2** with *R* = 0.996.

In summary, two new sandwich-type tetrakis(phthalocyaninato) dysprosium complexes with different extent of peripheral substitution on the phthalocyanine ligands have been prepared and structurally characterized. Comparative studies in their magnetic properties reveal the close relationship between the coordination geometry of the dysprosium spin carrier and the SMM behavior. This result is surely helpful for the design and synthesis of novel sandwich-type tetrakis(tetrapyrrole) lanthanide SMMs with their molecular structure and in turn magnetic properties optimized *through* simple tetrapyrrole peripheral substitution.

## Methods

### General remarks

1,2,4-Trichlorobenzene (TCB) and dichloromethane were freshly distilled from CaH_2_ under nitrogen. Column chromatography was carried out on silica gel columns (Merck, Kieselgel 60, 70–230 mesh) with the indicated eluents. All other reagents and solvents were used as received. The compounds of {(Pc)Dy[Pc(OC_5_H_11_)_8_]} and {[Pc(OC_5_H_11_)_8_]Dy[Pc(OC_5_H_11_)_8_]} were prepared according to the published procedure.

^1^H NMR spectra were recorded on a Bruker DPX 400 spectrometer in CDCl_3_. Spectrum was referenced internally using the residual solvent resonances (*δ* = 7.26 for ^1^H NMR). MALDI-TOF mass spectra were taken on a Bruker BIFLEX III ultrahighresolution Fourier transform ion cyclotron resonance (FT-ICR) mass spectrometer with alpha-cyano-4-hydroxycinnamic acid as matrix. Elemental analyses were performed on an Elementar Vavio El III.

### Single crystal X-ray diffraction determination

Data were collected on a Oxford Diffraction Gemini E diffractometer with Mo *Kα* radiation (λ = 0.7107 Å) at 120 K. Final unit cell parameters were derived by global refinements of reflections obtained from integration of all the frame data. The collected frames were integrated by using the preliminary cell-orientation matrix. CrysAlisPro Agilent Technologies software was used for collecting frames of data, indexing reflections, and determination of lattice constants and SCALE3 ABSPACK for absorption correction. The structures were solved by the direct method (*SHELXS-97*) and refined by full-matrix least-squares (*SHELXL-97*) on *F*^2^. Anisotropic thermal parameters were used for the nonhydrogen atoms and isotropic parameters for the hydrogen atoms. Hydrogen atoms were added geometrically and refined using a riding model. Crystallographic data and other pertinent information for the complex are summarized in Table S3 (Supplemental Information). Selected bond distances and bond angles with their estimated standard deviation are listed in Table S4 (Supplemental Information). CCDC 926978 for **1** and CCDC 994848 for **2** contain the supplementary crystallographic data for this paper. These data can be obtained free of charge from the Cambridge Crystallographic Data Centre *via* www.ccdc.cam.ac.uk/data_request/cif.

### Synthesis of 1 and 2

A mixture of neutral bis(phthalocyaninato) dysprosium compound {(Pc)Dy[Pc(OC_5_H_11_)_8_]} (37.4 mg, 0.02 mmol) and Cd(OAc)_2_. 2H_2_O (5.3 mg, 0.02 mmol) in TCB (2 mL) was heated to reflux under nitrogen for 3 h. After being cooled to room temperature, the volatiles were removed under reduced pressure. The residue was chromatographed on a silica gel column using CHCl_3_ as the eluent to give a green band, which contained mainly the unreacted bis(phthalocyaninato) dysprosium double-decker {(Pc)Dy[Pc(OC_5_H_11_)_8_]}. Further elution with CHCl_3_ gave a blue band containing the target quadruple-decker complex {(Pc)Dy[Pc(OC_5_H_11_)_8_]Cd[Pc(OC_5_H_11_)_8_]Dy(Pc)} (**1**), 27.2 mg (73%). In a similar manner with different double-decker as starting material, isolation of **2** in the yield of 61% was also achieved.

## Author Contributions

H.S., H.W. and J.J. designed the experiments and wrote the main paper; S.Z. and J.D. performed the magnetic measurements. All authors reviewed the manuscript.

## Additional Information

**Accession codes:** CCDC 926978 for **1** and CCDC 994848 for **2** contains the supplementary crystallographic data for this paper. These data can be obtained free of charge from The Cambridge Crystallographic Data Centre via www.ccdc.cam.ac.uk/data_request/cif.

## Supplementary Material

Supplementary Information supplementary information and checkcifs
