# Supplementary material for: Peripheral Substitution: An Easy Way to Tuning the Magnetic Behavior of Tetrakis(phthalocyaninato) Dysprosium(III) SMMs
Source: Sci Rep. 2015 Mar 6;5:8838. doi: 10.1038/srep08838 (PMC4351535; doi:10.1038/srep08838)
Supplement: Supplementary Information — and checkcifs [file srep08838-s1.pdf]

Supplementary Information

Peripheral Substitution: An Easy Way to Tuning the Magnetic Behavior of Tetrakis(phthalocyaninato) Dysprosium(III) SMMs

Hong Shang,[a] Suyuan Zeng,[b] Hailong Wang,[a] Jianmin Dou,*[b] & Jianzhuang Jiang*[a]

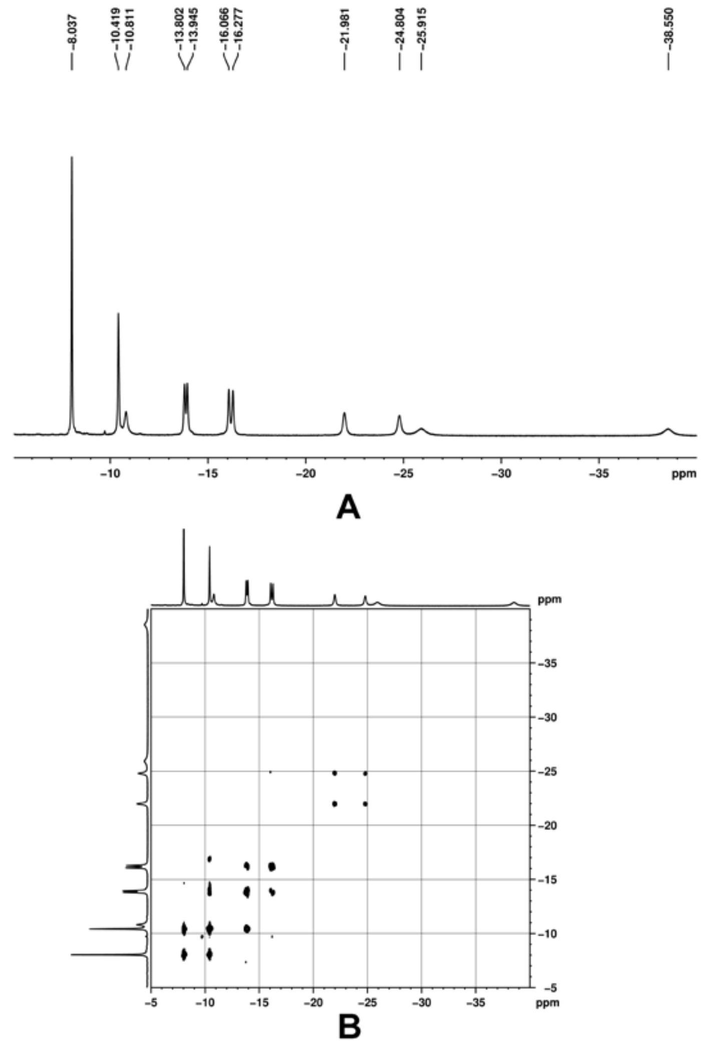

Figure S1. ^{1}H NMR and ^{1}H-^{1}H COSY spectra of {(Pc)Dy[Pc(OC_5H_{11})$_8$]Cd[Pc(OC_5H_{11})$_8$]Dy(Pc)} (**1**) in $CDCl_3$.

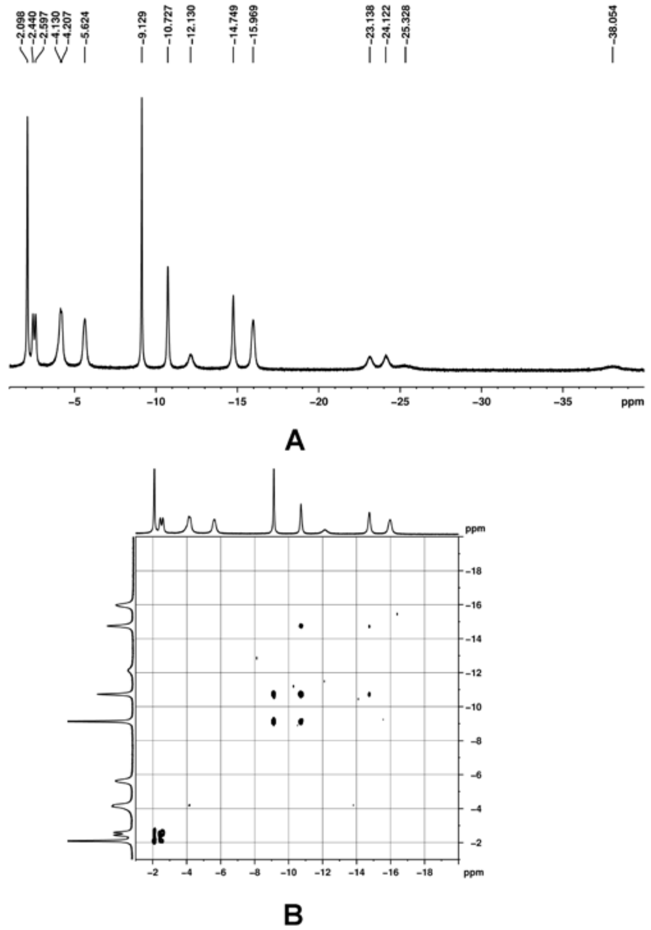

Figure S2. ^{1}H NMR and ^{1}H-^{1}H COSY spectra of {[Pc(OC_5H_{11})$_8$]Dy[Pc(OC_5H_{11})$_8$]Cd[Pc(OC_5H_{11})$_8$]Dy[Pc(OC_5H_{11})$_8$]} (**2**) in $CDCl_3$.

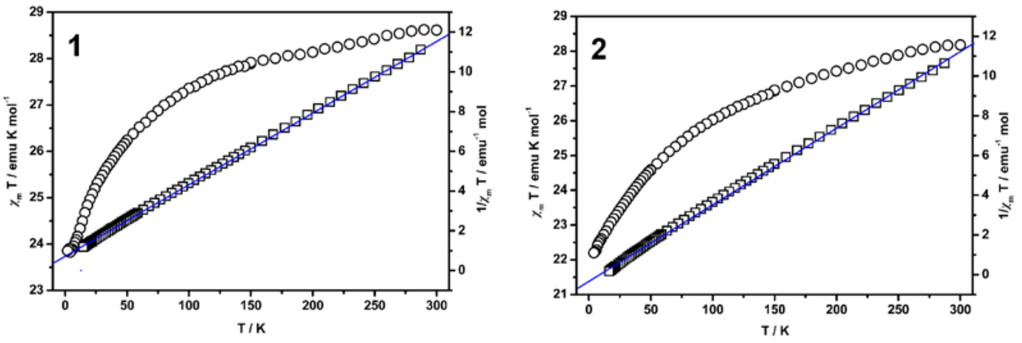

Figure S3. Temperature dependence of $\chi_m T$ for **1** and **2** (the blue solid line represents the best fitting for the complexes of 2-300 K).

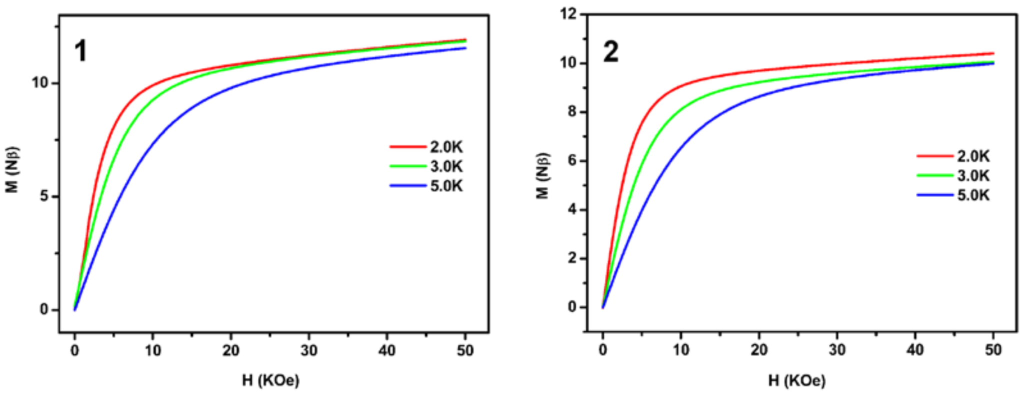

Figure S4. The *M vs H* curves for **1** and **2** at different temperature.

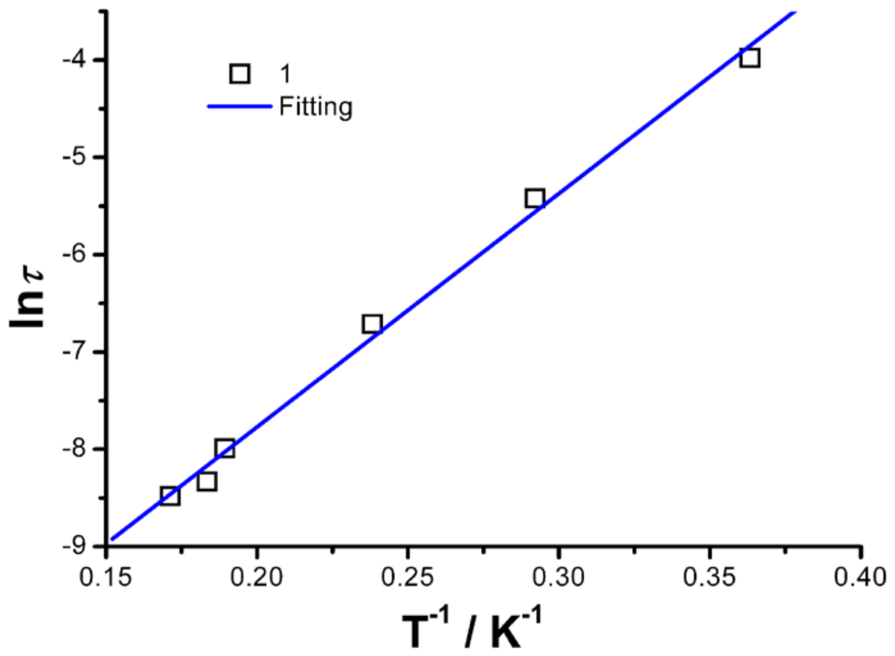

Figure S5. The plot of ln(τ) *vs*. 1/T for **1** under zero applied field.

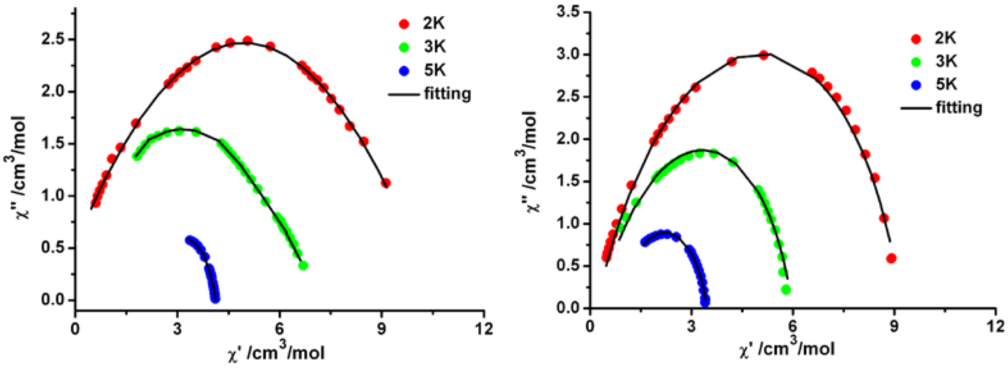

Figure S6. Cole-Cole diagrams of **1** (left) and **2** (right) with the ac susceptibility data under a zero applied dc field.

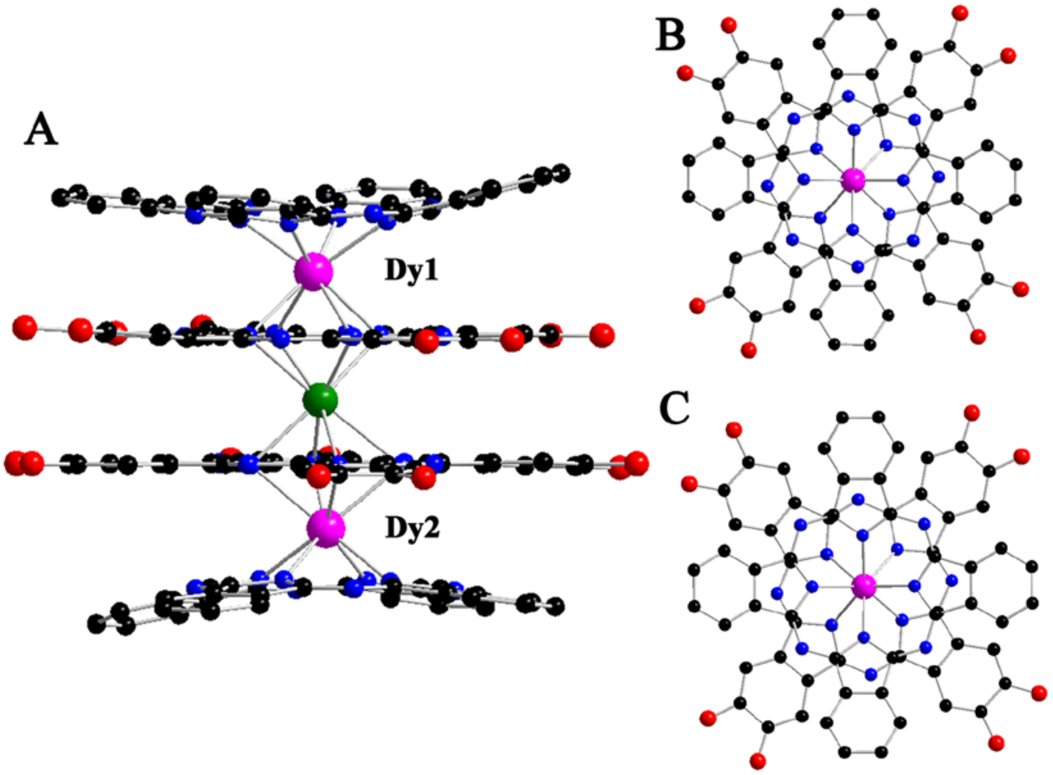

Figure S7. Molecular structures of **1** (A) in side view and Dy1 (B) and Dy2 (C) units in top view with all hydrogen atoms and C_5H_{11} side chains omitted for clarity [Dy(III) pink, Cd(II) green, C black, N blue, and O red].

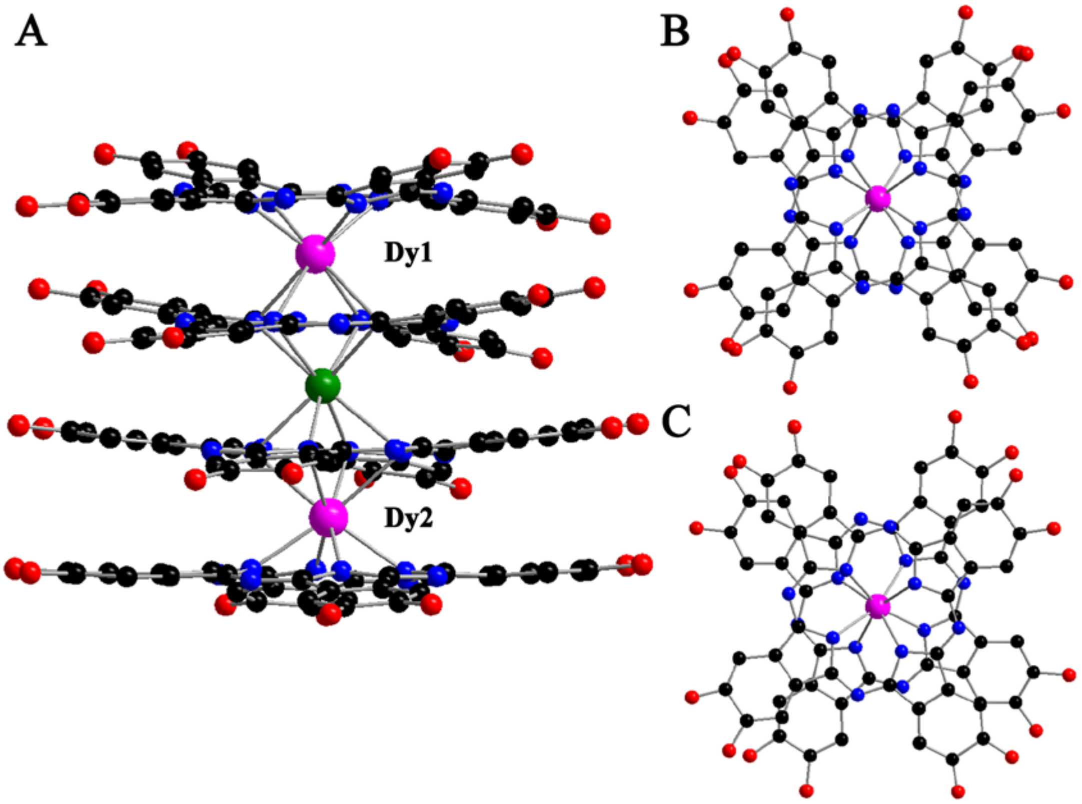

Figure S8. Molecular structures of **2** (A) in side view and Dy1 (B) and Dy2 (C) units in top view with all hydrogen atoms and C_5H_{11} side chains omitted for clarity [Dy(III) pink, Cd(II) green, C black, N blue, and O red].

Table S1. Mass spectroscopic and elemental analysis data for **1** and **2**.[a]

Compound	M^+ (*m/z*)[b]	Analysis (%)[a,b]		
		C	H	N
$C_{208}H_{224}CdDy_2N_{32}O_{16}$ (**1**)[c]	3868.9 (3865.6)	62.99 (64.63)	5.69 (5.84)	11.25 (11.59)
$C_{288}H_{384}CdDy_2N_{32}O_{32}$(**2**)	5237.1 (5243.7)	65.97 (65.97)	7.38 (7.38)	8.55 (8.55)

[a] Calculated values given in parentheses.[b] By MALDI-TOF mass spectrometry. The value corresponds to the most abundant isotopic peak of the protonated molecular ion. [c] Contain 1.0 equiv. of solvated $CHCl_3$.

Table S2. ^{1}H NMR data (δ) and assignments for **1** and **2** in $CDCl_3$.

Complex	H_α	H_β	-OC**H**$_2$-	-OCH$_2$(C**H**$_2$)$_3$-	-OCH$_2$(CH$_2$)$_3$C**H**$_3$
**1**	-38.55 (s, 16H)	-10.81(s, 16H)	-24.80 (d, 16H)	-16.28 (d, 16H)	-8.04 (s, 48H)
	-25.92 (s, 16H)		-21.98 (d, 16H)	-16.07 (d, 16H)	
				-13.95 (d, 16H)	
				-13.80 (d, 16H)	
				-10.81 (d, 16H)	
				-10.42 (s, 32H)	
**2**	-38.05 (s, 16H)		-24.12(d, 16H)	-14.75 (s, 32H)	-9.13 (s, 48H)
	-25.33 (s, 16H)		-23.14(d, 16H)	-15.97(s, 32H)	-2.10 (s, 48H)
			-12.13(s, 32H)	-10.73 (s, 32H)	
				-5.62 (s, 32H)	
				-4.13 (s, 32H)	
				-2.60 (d, 16H)	
				-2.44 (d, 16H)	

Table S3. Crystallographic data for **1** and **2**.

	**1**	**2**
Molecular formula	$C_{208}H_{224}CdDy_2N_{32}O_{16}$	$C_{288}H_{384}CdDy_2N_{32}O_{32}$
M	3865.60	5242.59
Crystal system	Monoclinic	Triclinic
Space group	C2/c	P -1
a/Å	27.2647(6)	23.6388(16)
b/Å	20.6335(4)	24.1129(13)
c/Å	37.2396(8)	29.0132(15)
α/°	90.00	74.965(5)
β/°	106.493(2)	79.072(5)
γ/°	90	68.413(6
U/Å^3	20087.7(7)	14769.3(15)
Z	4	2
D_c/Mg m^{-3}	1.436	1.179
μ/mm^{-1}	1.072	3.417
Data collection range/°	2.94 to 26.00	3.15 to 63.00
Reflections measured	77805	79507
Independent reflections	19703 (R_{int} = 0.0461)	47215 (R_{int} = 0.0508)
Parameters	126	3227
R_1 [$I>2\sigma(I)$]	0.0504	0.0919
wR_2 [$I>2\sigma(I)$]	0.0937	0.2440
Goodness of fit	1.101	0.865

Table S4. The structural data for **1** and **2**.

	**1**	**2**
average Dy-N(Pc’) bond distance (Å)	2.378	2.374
average Dy-N[Pc(OC_5H_{11})$_8$] bond distance (Å)	2.523	2.558
average Cd-N[Pc(OC_5H_{11})$_8$] bond distance (Å)	2.538	2.527
Dy-N_4(Pc’) plane distance (Å)	1.326	1.321
Dy-N_4[Pc(OC_5H_{11})$_8$)] plane distance (Å)	1.621	1.681
interplanar distance (Å)	3.276	3.260
dihedral angle between the N_4 planes for (Pc’)N_4-Dy-N_4[Pc(OC_5H_{11})$_8$] (^{o})	0.889	0.945
dihedral angle between the N_4 planes for [Pc(OC_5H_{11})$_8$]N_4-Cd-N_4[Pc(OC_5H_{11})$_8$] (^{o})	0.493	0.712
average dihedral angle φ for the Pc’ ring (^{o})[a]	9.379	7.715
average dihedral angle φ for the Pc(OC_5H_{11})$_8$ ring (^{o})[a]	1.673	6.375
average twist angle for (Pc’)N_4-Dy-N_4[Pc(OC_5H_{11})$_8$] (^{o})[b]	41.418	23.460
average twist angle for [Pc(OC_5H_{11})$_8$]N_4-Cd-N_4[Pc(OC_5H_{11})$_8$] (^{o})	21.315	20.103
nearest inter-molucular Dy···Dy distance (Å)	12.755	13.743

[a] The average dihedral angle of the individual isoindole rings with respect to the corresponding N_4 mean plane. [b] Defined as the

rotation angle of one macrocycle away from the eclipsed conformation of the two macrocycles.

checkCIF/PLATON report

You have not supplied any structure factors. As a result the full set of tests cannot be run.

THIS REPORT IS FOR GUIDANCE ONLY. IF USED AS PART OF A REVIEW PROCEDURE FOR PUBLICATION, IT SHOULD NOT REPLACE THE EXPERTISE OF AN EXPERIENCED CRYSTALLOGRAPHIC REFEREE.

No syntax errors found. CIF dictionary Interpreting this report

Datablock: 1

Bond precision: C-C = 0.0060 A Wavelength=0.71073

Cell:	a=27.2647(6)	b=20.6335(4)	c=37.2396(8)
	alpha=90	beta=106.493(2)	gamma=90
Temperature:	120 K		

	Calculated	Reported
Volume	20087.8(8)	20087.7(7)
Space group	C 2/c	C 1 2/c 1
Hall group	-C 2yc	?
Moiety formula	C208 H224 Cd Dy2 N32 O16, 4(C H Cl3)	?
Sum formula	C212 H228 Cd Cl12 Dy2 N32 O16	C106 H114 Cd0.50 Cl6 Dy N16 O8
Mr	4343.09	2171.53
Dx,g cm-3	1.436	1.436
Z	4	8
Mu (mm-1)	1.072	1.072
F000	8944.0	8944.0
F000’	8949.09	
h,k,lmax	33,25,45	33,25,45
Nref	19741	19703
Tmin,Tmax	0.902,0.948	0.894,1.000
Tmin’	0.898	

Correction method= MULTI-SCAN

Data completeness= 0.998 Theta(max)= 26.000

R(reflections)= 0.0504(15448) wR2(reflections)= 0.0995(19703)

S = 1.106 Npar= 1261

```
The following ALERTS were generated. Each ALERT has the format
       test-name_ALERT_alert-type_alert-level.
Click on the hyperlinks for more details of the test.
```

Alert level A

```
PLAT410_ALERT_2_A Short Intra H...H Contact  H66A   ..  H67D    ..       1.70 Ang.
```

Alert level B

```
PLAT220_ALERT_2_B Large Non-Solvent  C     Ueq(max)/Ueq(min) Range        7.0 Ratio
```

Alert level C

```
PLAT222_ALERT_3_C Large Non-Solvent    H    Uiso(max)/Uiso(min) ..        6.6 Ratio
PLAT244_ALERT_4_C Low   'Solvent' Ueq as Compared to Neighbors of        C105 Check
PLAT413_ALERT_2_C Short Inter XH3 .. XHn     H13    ..  H69E    ..       2.11 Ang.
```

Alert level G

```
PLAT002_ALERT_2_G Number of Distance or Angle Restraints on AtSite          8 Note
PLAT003_ALERT_2_G Number of Uiso or Uij Restrained non-H Atoms ...        141 Report
PLAT005_ALERT_5_G No _iucr_refine_instructions_details  in the CIF     Please Do !
PLAT045_ALERT_1_G Calculated and Reported Z Differ by ............       0.50 Ratio
PLAT083_ALERT_2_G SHELXL Second Parameter in WGHT Unusually Large.     175.03 Why ?
PLAT301_ALERT_3_G Main Residue  Disorder ............ Percentage =          2 Note
PLAT793_ALERT_4_G The Model has Chirality at N9      .............          R Verify
PLAT793_ALERT_4_G The Model has Chirality at N11     .............          R Verify
PLAT793_ALERT_4_G The Model has Chirality at N13     .............          R Verify
PLAT793_ALERT_4_G The Model has Chirality at N15     .............          S Verify
PLAT860_ALERT_3_G Number of Least-Squares Restraints .............       3816 Note
PLAT899_ALERT_4_G SHELXL97   is Deprecated and Succeeded by SHELXL       2014 Note
```

```
   1 ALERT level A = Most likely a serious problem - resolve or explain
   1 ALERT level B = A potentially serious problem, consider carefully
   3 ALERT level C = Check. Ensure it is not caused by an omission or oversight
  12 ALERT level G = General information/check it is not something unexpected

   1 ALERT type 1 CIF construction/syntax error, inconsistent or missing data
   6 ALERT type 2 Indicator that the structure model may be wrong or deficient
   3 ALERT type 3 Indicator that the structure quality may be low
   6 ALERT type 4 Improvement, methodology, query or suggestion
   1 ALERT type 5 Informative message, check
```

It is advisable to attempt to resolve as many as possible of the alerts in all categories. Often the minor alerts point to easily fixed oversights, errors and omissions in your CIF or refinement strategy, so attention to these fine details can be worthwhile. In order to resolve some of the more serious problems it may be necessary to carry out additional measurements or structure refinements. However, the purpose of your study may justify the reported deviations and the more serious of these should normally be commented upon in the discussion or experimental section of a paper or in the "special_details" fields of the CIF. checkCIF was carefully designed to identify outliers and unusual parameters, but every test has its limitations and alerts that are not important in a particular case may appear. Conversely, the absence of alerts does not guarantee there are no aspects of the results needing attention. It is up to the individual to critically assess their own results and, if necessary, seek expert advice.

Publication of your CIF in IUCr journals

A basic structural check has been run on your CIF. These basic checks will be run on all CIFs submitted for publication in IUCr journals (*Acta Crystallographica*, *Journal of Applied Crystallography*, *Journal of Synchrotron Radiation*); however, if you intend to submit to *Acta Crystallographica Section C* or *E*, you should make sure that full publication checks are run on the final version of your CIF prior to submission.

Publication of your CIF in other journals

Please refer to the *Notes for Authors* of the relevant journal for any special instructions relating to CIF submission.

PLATON version of 20/08/2014; check.def file version of 18/08/2014

Datablock 1 - ellipsoid plot

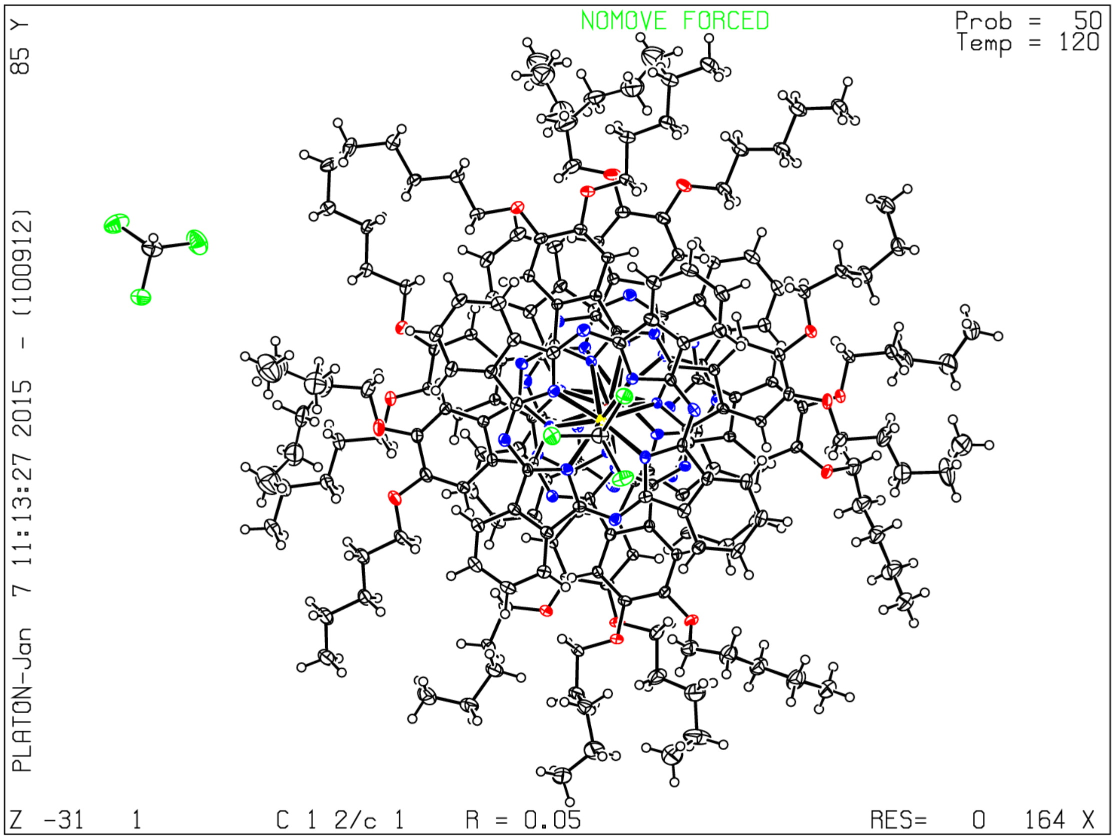

A Alert

Short Intra H...H Contact H66A .. H67D .. 1.70 Ang.

Explain

The A Alert in the present case is actually due to the disorder of the related alky chain including C67 C68 C69 and C67A C68A C69A, which leads to a short intra H...H contact between H66A and H67D.

checkCIF/PLATON report

You have not supplied any structure factors. As a result the full set of tests cannot be run.

THIS REPORT IS FOR GUIDANCE ONLY. IF USED AS PART OF A REVIEW PROCEDURE FOR PUBLICATION, IT SHOULD NOT REPLACE THE EXPERTISE OF AN EXPERIENCED CRYSTALLOGRAPHIC REFEREE.

No syntax errors found. CIF dictionary Interpreting this report

Datablock: 2

```
Bond precision:  C-C = 0.0173 A          Wavelength=1.54184

Cell:          a=23.6388(16)      b=24.1129(13)     c=29.0132(15)
               alpha=74.965(5)    beta=79.072(5)    gamma=68.413(6)
Temperature:   150 K

                Calculated                Reported
Volume          14769.4(16)               14769.3(15)
Space group     P -1                      P -1
Hall group      -P 1                      ?
Moiety formula  C288 H383 Cd Dy2 N32 O32  ?
Sum formula     C288 H383 Cd Dy2 N32 O32  C288 H383 Cd Dy2 N32 O32
Mr              5242.59                   5242.59
Dx,g cm-3       1.179                     1.179
Z               2                         2
Mu (mm-1)       3.769                     3.769
F000            5542.0                    5542.0
F000'           5517.87
h,k,lmax        27,27,33                  27,27,33
Nref            47755                     47215
Tmin,Tmax       0.668,0.740               0.310,1.000
Tmin'           0.541

Correction method= MULTI-SCAN

Data completeness= 0.989          Theta(max)= 63.000

R(reflections)= 0.0919( 24255)    wR2(reflections)= 0.2759( 47215)

S = 0.865                 Npar= 3227
```

The following ALERTS were generated. Each ALERT has the format
test-name_ALERT_alert-type_alert-level.
Click on the hyperlinks for more details of the test.

Alert level A

```
PLAT410_ALERT_2_A Short Intra H...H Contact  H13O   ..  H15N    ..        0.94 Ang.
PLAT410_ALERT_2_A Short Intra H...H Contact  H15V   ..  H92B    ..        1.70 Ang.
PLAT411_ALERT_2_A Short Inter H...H Contact  H1W1   ..  H11     ..        1.56 Ang.
PLAT411_ALERT_2_A Short Inter H...H Contact  H1W1   ..  H11W    ..        0.79 Ang.
PLAT411_ALERT_2_A Short Inter H...H Contact  H10I   ..  H89B    ..        1.51 Ang.
PLAT411_ALERT_2_A Short Inter H...H Contact  H10M   ..  H87B    ..        1.70 Ang.
PLAT411_ALERT_2_A Short Inter H...H Contact  H10N   ..  H87A    ..        1.75 Ang.
PLAT412_ALERT_2_A Short Intra XH3 .. XHn     H14L   ..  H16D    ..        1.32 Ang.
PLAT412_ALERT_2_A Short Intra XH3 .. XHn     H14M   ..  H16C    ..        1.55 Ang.
PLAT412_ALERT_2_A Short Intra XH3 .. XHn     H14M   ..  H16D    ..        1.67 Ang.
PLAT412_ALERT_2_A Short Intra XH3 .. XHn     H34B   ..  H95B    ..        1.44 Ang.
PLAT412_ALERT_2_A Short Intra XH3 .. XHn     H65B   ..  H95A    ..        1.61 Ang.
PLAT413_ALERT_2_A Short Inter XH3 .. XHn     H10L   ..  H15F    ..        1.78 Ang.
PLAT413_ALERT_2_A Short Inter XH3 .. XHn     H10Q   ..  H85A    ..        1.82 Ang.
PLAT413_ALERT_2_A Short Inter XH3 .. XHn     H13C   ..  H98A    ..        1.85 Ang.
PLAT413_ALERT_2_A Short Inter XH3 .. XHn     H35B   ..  H35B    ..        1.69 Ang.
PLAT413_ALERT_2_A Short Inter XH3 .. XHn     H75C   ..  H85B    ..        1.89 Ang.
PLAT413_ALERT_2_A Short Inter XH3 .. XHn     H75C   ..  H85C    ..        1.61 Ang.
```

Alert level B

```
PLAT220_ALERT_2_B Large Non-Solvent  C     Ueq(max)/Ueq(min) Range        6.6 Ratio
PLAT222_ALERT_3_B Large Non-Solvent    H    Uiso(max)/Uiso(min) ..        7.5 Ratio
PLAT230_ALERT_2_B Hirshfeld Test Diff for    O5D    --  C141    ..         9.3 su
PLAT230_ALERT_2_B Hirshfeld Test Diff for    O7A    --  C31     ..         7.1 su
PLAT230_ALERT_2_B Hirshfeld Test Diff for    O8D    --  C156    ..         8.3 su
PLAT230_ALERT_2_B Hirshfeld Test Diff for    C67    --  C68     ..         8.1 su
PLAT230_ALERT_2_B Hirshfeld Test Diff for    C72    --  C73     ..         8.0 su
PLAT230_ALERT_2_B Hirshfeld Test Diff for    C86    --  C87     ..         8.0 su
PLAT230_ALERT_2_B Hirshfeld Test Diff for    C132   --  C133    ..        11.6 su
PLAT230_ALERT_2_B Hirshfeld Test Diff for    C149   --  C150    ..        14.3 su
PLAT234_ALERT_4_B Large Hirshfeld Difference C8     --  C9      ..        0.28 Ang.
PLAT234_ALERT_4_B Large Hirshfeld Difference C9     --  C10     ..        0.26 Ang.
PLAT234_ALERT_4_B Large Hirshfeld Difference C64    --  C65     ..        0.26 Ang.
PLAT234_ALERT_4_B Large Hirshfeld Difference C69    --  C70     ..        0.26 Ang.
PLAT234_ALERT_4_B Large Hirshfeld Difference C74    --  C75     ..        0.26 Ang.
PLAT234_ALERT_4_B Large Hirshfeld Difference C94    --  C95     ..        0.27 Ang.
PLAT234_ALERT_4_B Large Hirshfeld Difference C139   --  C140    ..        0.26 Ang.
PLAT242_ALERT_2_B Low       Ueq as Compared to Neighbors for .....        C109 Check
PLAT351_ALERT_3_B Long    C-H (X0.96,N1.08A) C135   -   H15P_a ...        1.26 Ang.
PLAT351_ALERT_3_B Long    C-H (X0.96,N1.08A) C145   -   H16C_c ...        1.21 Ang.
PLAT410_ALERT_2_B Short Intra H...H Contact  H14G   ..  H1F4    ..        1.89 Ang.
PLAT411_ALERT_2_B Short Inter H...H Contact  H10J   ..  H89A    ..        1.81 Ang.
PLAT412_ALERT_2_B Short Intra XH3 .. XHn     H12B   ..  H15A    ..        1.71 Ang.
PLAT412_ALERT_2_B Short Intra XH3 .. XHn     H14L   ..  H16C    ..        1.73 Ang.
PLAT412_ALERT_2_B Short Intra XH3 .. XHn     H35A   ..  H80A    ..        1.74 Ang.
PLAT413_ALERT_2_B Short Inter XH3 .. XHn     H2B    ..  H80C    ..        1.93 Ang.
PLAT413_ALERT_2_B Short Inter XH3 .. XHn     H10P   ..  H83B    ..        1.96 Ang.
PLAT413_ALERT_2_B Short Inter XH3 .. XHn     H54A   ..  H80B    ..        1.94 Ang.
```

Alert level C

```
RFACR01_ALERT_3_C  The value of the weighted R factor is > 0.25
            Weighted R factor given   0.276
THETM01_ALERT_3_C  The value of sine(theta_max)/wavelength is less than 0.590
            Calculated sin(theta_max)/wavelength =    0.5779
PLAT084_ALERT_3_C High wR2 Value (i.e. > 0.25) ...................        0.28 Report
PLAT230_ALERT_2_C Hirshfeld Test Diff for    O5C    --  C101    ..         6.8 su
PLAT230_ALERT_2_C Hirshfeld Test Diff for    C25D   --  C26D    ..         6.0 su
PLAT230_ALERT_2_C Hirshfeld Test Diff for    C34    --  C35     ..         5.5 su
```

```
PLAT230_ALERT_2_C Hirshfeld Test Diff for    C51    --  C52     ..        6.0 su
PLAT230_ALERT_2_C Hirshfeld Test Diff for    C84    --  C85     ..        7.0 su
PLAT230_ALERT_2_C Hirshfeld Test Diff for    C101   --  C102    ..        5.4 su
PLAT230_ALERT_2_C Hirshfeld Test Diff for    C109   --  C110    ..        5.5 su
PLAT230_ALERT_2_C Hirshfeld Test Diff for    C116   --  C117    ..        7.0 su
PLAT230_ALERT_2_C Hirshfeld Test Diff for    C128   --  C129    ..        5.2 su
PLAT230_ALERT_2_C Hirshfeld Test Diff for    C137   --  C138    ..        5.8 su
PLAT230_ALERT_2_C Hirshfeld Test Diff for    C144   --  C145    ..        6.5 su
PLAT234_ALERT_4_C Large Hirshfeld Difference O2A    --  C20A    ..       0.16 Ang.
PLAT234_ALERT_4_C Large Hirshfeld Difference O2B    --  C46     ..       0.16 Ang.
PLAT234_ALERT_4_C Large Hirshfeld Difference C1     --  C2      ..       0.18 Ang.
PLAT234_ALERT_4_C Large Hirshfeld Difference C11C   --  C12C    ..       0.16 Ang.
PLAT234_ALERT_4_C Large Hirshfeld Difference C14    --  C15     ..       0.21 Ang.
PLAT234_ALERT_4_C Large Hirshfeld Difference C18    --  C19     ..       0.18 Ang.
PLAT234_ALERT_4_C Large Hirshfeld Difference C23    --  C24     ..       0.16 Ang.
PLAT234_ALERT_4_C Large Hirshfeld Difference C31    --  C32     ..       0.17 Ang.
PLAT234_ALERT_4_C Large Hirshfeld Difference C32    --  C33     ..       0.20 Ang.
PLAT234_ALERT_4_C Large Hirshfeld Difference C44    --  C45     ..       0.24 Ang.
PLAT234_ALERT_4_C Large Hirshfeld Difference C53    --  C54     ..       0.25 Ang.
PLAT234_ALERT_4_C Large Hirshfeld Difference C54    --  C55     ..       0.22 Ang.
PLAT234_ALERT_4_C Large Hirshfeld Difference C58    --  C59     ..       0.18 Ang.
PLAT234_ALERT_4_C Large Hirshfeld Difference C59    --  C60     ..       0.22 Ang.
PLAT234_ALERT_4_C Large Hirshfeld Difference C71    --  C72     ..       0.17 Ang.
PLAT234_ALERT_4_C Large Hirshfeld Difference C73    --  C74     ..       0.19 Ang.
PLAT234_ALERT_4_C Large Hirshfeld Difference C78    --  C79     ..       0.18 Ang.
PLAT234_ALERT_4_C Large Hirshfeld Difference C88    --  C89     ..       0.24 Ang.
PLAT234_ALERT_4_C Large Hirshfeld Difference C92    --  C93     ..       0.17 Ang.
PLAT234_ALERT_4_C Large Hirshfeld Difference C93    --  C94     ..       0.20 Ang.
PLAT234_ALERT_4_C Large Hirshfeld Difference C98    --  C99     ..       0.24 Ang.
PLAT234_ALERT_4_C Large Hirshfeld Difference C103   --  C104    ..       0.18 Ang.
PLAT234_ALERT_4_C Large Hirshfeld Difference C112   --  C113    ..       0.22 Ang.
PLAT234_ALERT_4_C Large Hirshfeld Difference C118   --  C119    ..       0.22 Ang.
PLAT234_ALERT_4_C Large Hirshfeld Difference C124   --  C125    ..       0.20 Ang.
PLAT234_ALERT_4_C Large Hirshfeld Difference C127   --  C128    ..       0.20 Ang.
PLAT234_ALERT_4_C Large Hirshfeld Difference C129   --  C130    ..       0.24 Ang.
PLAT234_ALERT_4_C Large Hirshfeld Difference C134   --  C135    ..       0.24 Ang.
PLAT234_ALERT_4_C Large Hirshfeld Difference C138   --  C139    ..       0.17 Ang.
PLAT234_ALERT_4_C Large Hirshfeld Difference C141   --  C142    ..       0.20 Ang.
PLAT234_ALERT_4_C Large Hirshfeld Difference C152   --  C153    ..       0.17 Ang.
PLAT234_ALERT_4_C Large Hirshfeld Difference C154   --  C155    ..       0.22 Ang.
PLAT234_ALERT_4_C Large Hirshfeld Difference C157   --  C158    ..       0.18 Ang.
PLAT234_ALERT_4_C Large Hirshfeld Difference C158   --  C159    ..       0.24 Ang.
PLAT241_ALERT_2_C High      Ueq as Compared to Neighbors for .....        C69 Check
PLAT241_ALERT_2_C High      Ueq as Compared to Neighbors for .....        C87 Check
PLAT241_ALERT_2_C High      Ueq as Compared to Neighbors for .....       C117 Check
PLAT241_ALERT_2_C High      Ueq as Compared to Neighbors for .....       C138 Check
PLAT241_ALERT_2_C High      Ueq as Compared to Neighbors for .....       C142 Check
PLAT242_ALERT_2_C Low       Ueq as Compared to Neighbors for .....        O5D Check
PLAT242_ALERT_2_C Low       Ueq as Compared to Neighbors for .....         C4 Check
PLAT242_ALERT_2_C Low       Ueq as Compared to Neighbors for .....        C16 Check
PLAT242_ALERT_2_C Low       Ueq as Compared to Neighbors for .....        C18 Check
PLAT242_ALERT_2_C Low       Ueq as Compared to Neighbors for .....        C29 Check
PLAT242_ALERT_2_C Low       Ueq as Compared to Neighbors for .....        C34 Check
PLAT242_ALERT_2_C Low       Ueq as Compared to Neighbors for .....        C41 Check
PLAT242_ALERT_2_C Low       Ueq as Compared to Neighbors for .....        C44 Check
PLAT242_ALERT_2_C Low       Ueq as Compared to Neighbors for .....        C51 Check
PLAT242_ALERT_2_C Low       Ueq as Compared to Neighbors for .....        C59 Check
PLAT242_ALERT_2_C Low       Ueq as Compared to Neighbors for .....        C74 Check
PLAT242_ALERT_2_C Low       Ueq as Compared to Neighbors for .....        C79 Check
PLAT242_ALERT_2_C Low       Ueq as Compared to Neighbors for .....        C84 Check
PLAT242_ALERT_2_C Low       Ueq as Compared to Neighbors for .....        C86 Check
PLAT242_ALERT_2_C Low       Ueq as Compared to Neighbors for .....       C116 Check
```

```
PLAT242_ALERT_2_C Low       Ueq as Compared to Neighbors for .....       C118 Check
PLAT242_ALERT_2_C Low       Ueq as Compared to Neighbors for .....       C119 Check
PLAT242_ALERT_2_C Low       Ueq as Compared to Neighbors for .....       C124 Check
PLAT242_ALERT_2_C Low       Ueq as Compared to Neighbors for .....       C129 Check
PLAT242_ALERT_2_C Low       Ueq as Compared to Neighbors for .....       C132 Check
PLAT242_ALERT_2_C Low       Ueq as Compared to Neighbors for .....       C139 Check
PLAT242_ALERT_2_C Low       Ueq as Compared to Neighbors for .....       C144 Check
PLAT242_ALERT_2_C Low       Ueq as Compared to Neighbors for .....       C149 Check
PLAT303_ALERT_2_C Full Occupancy H-Atom  H13N   with # Connections       2.00 Check
PLAT303_ALERT_2_C Full Occupancy H-Atom  H15P   with # Connections       2.00 Check
PLAT303_ALERT_2_C Full Occupancy H-Atom  H16C   with # Connections       2.00 Check
PLAT342_ALERT_3_C Low Bond Precision on  C-C Bonds ...............     0.0173 Ang.
PLAT351_ALERT_3_C Long    C-H (X0.96,N1.08A) C155   -   H13N_b ...       1.12 Ang.
PLAT410_ALERT_2_C Short Intra H...H Contact  H3B1   ..  H71      ..      1.97 Ang.
PLAT410_ALERT_2_C Short Intra H...H Contact  H13N   ..  H15N     ..      1.94 Ang.
PLAT410_ALERT_2_C Short Intra H...H Contact  H14P   ..  H15W     ..      1.92 Ang.
PLAT411_ALERT_2_C Short Inter H...H Contact  H5W5   ..  H11      ..      2.11 Ang.
PLAT411_ALERT_2_C Short Inter H...H Contact  H10N   ..  H87B     ..      2.09 Ang.
PLAT411_ALERT_2_C Short Inter H...H Contact  H10N   ..  H89B     ..      2.00 Ang.
PLAT411_ALERT_2_C Short Inter H...H Contact  H13K   ..  H13T     ..      2.01 Ang.
PLAT412_ALERT_2_C Short Intra XH3 .. XHn     H14N   ..  H16E     ..      1.83 Ang.
PLAT413_ALERT_2_C Short Inter XH3 .. XHn     H10B   ..  H35C     ..      2.14 Ang.
PLAT413_ALERT_2_C Short Inter XH3 .. XHn     H12C   ..  H98B     ..      2.11 Ang.
PLAT413_ALERT_2_C Short Inter XH3 .. XHn     H12E   ..  H79B     ..      2.00 Ang.
PLAT413_ALERT_2_C Short Inter XH3 .. XHn     H16E   ..  H54B     ..      2.04 Ang.
```

Alert level G

```
PLAT002_ALERT_2_G Number of Distance or Angle Restraints on AtSite        171 Note
PLAT003_ALERT_2_G Number of Uiso or Uij Restrained non-H Atoms ...        355 Report
PLAT004_ALERT_5_G Polymeric Structure Found with Dimension .......          1 Info
PLAT005_ALERT_5_G No _iucr_refine_instructions_details  in the CIF     Please Do !
PLAT072_ALERT_2_G SHELXL First  Parameter in WGHT Unusually Large.       0.19 Report
PLAT232_ALERT_2_G Hirshfeld Test Diff (M-X)  Dy1    --  N7B      ..       5.3 su
PLAT232_ALERT_2_G Hirshfeld Test Diff (M-X)  Dy2    --  N7C      ..       6.3 su
PLAT335_ALERT_2_G Check Large C6 Ring    C-C Range C2C    -C7C           0.16 Ang.
PLAT343_ALERT_2_G Unusual          Angle Range in Main Residue for        C135   Che
PLAT343_ALERT_2_G Unusual          Angle Range in Main Residue for        C145   Che
PLAT343_ALERT_2_G Unusual          Angle Range in Main Residue for        C155   Che
PLAT432_ALERT_2_G Short Inter X...Y Contact  C75    ..  C85      ..      3.01 Ang.
PLAT432_ALERT_2_G Short Inter X...Y Contact  C87    ..  C104     ..      2.77 Ang.
PLAT432_ALERT_2_G Short Inter X...Y Contact  C89    ..  C102     ..      2.71 Ang.
PLAT432_ALERT_2_G Short Inter X...Y Contact  C89    ..  C104     ..      3.18 Ang.
PLAT432_ALERT_2_G Short Inter X...Y Contact  C119   ..  C159     ..      2.35 Ang.
PLAT432_ALERT_2_G Short Inter X...Y Contact  C119   ..  C158     ..      3.17 Ang.
PLAT605_ALERT_4_G Structure Contains Solvent Accessible VOIDS of .        475 A**3
PLAT720_ALERT_4_G Number of Unusual/Non-Standard Labels ..........         16 Note
PLAT773_ALERT_2_G Check long C-C Bond in CIF: C144   --  C160    .       1.96 Ang.
PLAT773_ALERT_2_G Check long C-C Bond in CIF: C160   --  C144    .       1.96 Ang.
PLAT793_ALERT_4_G The Model has Chirality at N1B     .............          S Verify
PLAT793_ALERT_4_G The Model has Chirality at N1C     .............          R Verify
PLAT793_ALERT_4_G The Model has Chirality at N3B     .............          S Verify
PLAT793_ALERT_4_G The Model has Chirality at N3C     .............          R Verify
PLAT793_ALERT_4_G The Model has Chirality at N5B     .............          R Verify
PLAT793_ALERT_4_G The Model has Chirality at N5C     .............          R Verify
PLAT793_ALERT_4_G The Model has Chirality at N7B     .............          R Verify
PLAT793_ALERT_4_G The Model has Chirality at N7C     .............          S Verify
PLAT860_ALERT_3_G Number of Least-Squares Restraints .............      12182 Note
PLAT869_ALERT_4_G ALERTS Related to the use of SQUEEZE Suppressed           ! Info
PLAT899_ALERT_4_G SHELXL97   is Deprecated and Succeeded by SHELXL       2014 Note
PLAT982_ALERT_1_G The Dy-f'=    -9.147 Deviates from the  IT-value     -9.805 Check
```

```
PLAT983_ALERT_1_G The Dy-f"=     9.189 Deviates from the  IT-Value      9.848 Check
```

```
  18 ALERT level A = Most likely a serious problem - resolve or explain
  28 ALERT level B = A potentially serious problem, consider carefully
  93 ALERT level C = Check. Ensure it is not caused by an omission or oversight
  34 ALERT level G = General information/check it is not something unexpected

   2 ALERT type 1 CIF construction/syntax error, inconsistent or missing data
 107 ALERT type 2 Indicator that the structure model may be wrong or deficient
   9 ALERT type 3 Indicator that the structure quality may be low
  53 ALERT type 4 Improvement, methodology, query or suggestion
   2 ALERT type 5 Informative message, check
```

It is advisable to attempt to resolve as many as possible of the alerts in all categories. Often the minor alerts point to easily fixed oversights, errors and omissions in your CIF or refinement strategy, so attention to these fine details can be worthwhile. In order to resolve some of the more serious problems it may be necessary to carry out additional measurements or structure refinements. However, the purpose of your study may justify the reported deviations and the more serious of these should normally be commented upon in the discussion or experimental section of a paper or in the "special_details" fields of the CIF. checkCIF was carefully designed to identify outliers and unusual parameters, but every test has its limitations and alerts that are not important in a particular case may appear. Conversely, the absence of alerts does not guarantee there are no aspects of the results needing attention. It is up to the individual to critically assess their own results and, if necessary, seek expert advice.

Publication of your CIF in IUCr journals

A basic structural check has been run on your CIF. These basic checks will be run on all CIFs submitted for publication in IUCr journals (*Acta Crystallographica*, *Journal of Applied Crystallography*, *Journal of Synchrotron Radiation*); however, if you intend to submit to *Acta Crystallographica Section C* or *E*, you should make sure that full publication checks are run on the final version of your CIF prior to submission.

Publication of your CIF in other journals

Please refer to the *Notes for Authors* of the relevant journal for any special instructions relating to CIF submission.

PLATON version of 20/08/2014; check.def file version of 18/08/2014

Datablock 2 - ellipsoid plot

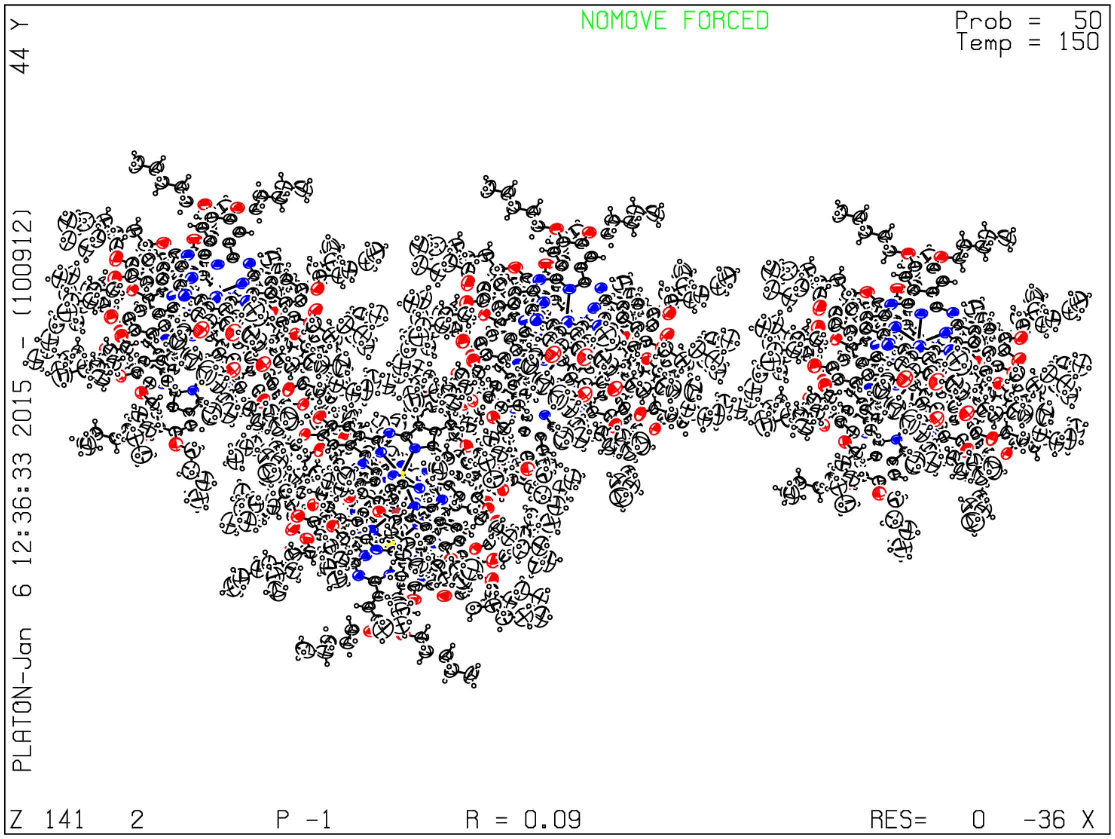

A Alert

(1) Short Intra H...H Contact H13O .. H15N .. 0.94 Ang.
(2) Short Intra H...H Contact H15V .. H92B .. 1.70 Ang.
(3) Short Inter H...H Contact H1W1 .. H11 .. 1.56 Ang.
(4) Short Inter H...H Contact H1W1 .. H11W .. 0.79 Ang.
(5) Short Inter H...H Contact H10I .. H89B .. 1.51 Ang.
(6) Short Inter H...H Contact H10M .. H87B .. 1.70 Ang.
(7) Short Inter H...H Contact H10N .. H87A .. 1.75 Ang.
(8) Short Intra XH3 .. XHn H14L .. H16D .. 1.32 Ang.
(9) Short Intra XH3 .. XHn H14M .. H16C .. 1.55 Ang.
(10) Short Intra XH3 .. XHn H14M .. H16D .. 1.67 Ang.
(11) Short Intra XH3 .. XHn H34B .. H95B .. 1.44 Ang.
(12) Short Intra XH3 .. XHn H65B .. H95A .. 1.61 Ang.
(13) Short Inter XH3 .. XHn H10L .. H15F .. 1.78 Ang.
(14) Short Inter XH3 .. XHn H10Q .. H85A .. 1.82 Ang.
(15) Short Inter XH3 .. XHn H13C .. H98A .. 1.85 Ang.
(16) Short Inter XH3 .. XHn H35B .. H35B .. 1.69 Ang.
(17) Short Inter XH3 .. XHn H75C .. H85B .. 1.89 Ang.
(18) Short Inter XH3 .. XHn H75C .. H85C .. 1.61 Ang.

Explain

The A Alert in the present case are actually due to the inherent weak diffraction of the single crystal of this compound associated with the large number of carbon atoms, which hampers the optimization of the calculated H atoms position.

In the present structure, the unit cell includes a large region of disordered solvent molecules, which could not be modeled as discrete atomic sites. We employed PLATON/SQUEEZE to calculate the diffraction contribution of the solvent molecules and, thereby, to produce a set of solvent-free diffraction intensities. For this structure, the SQUEEZE calculations showed a total solvent accessible area volume of 4801 $Å^3$ and the residual electron density amounted to 1021 electron per unit cell, corresponding to nearly 12 molecules of chloroform and 24 molecules of methanol (about 6 chloroform and 12 methanol molecules per asymmetric unit).
